# Effects of dalfampridine and its metabolites on cloned human potassium channels K_v_ 1.1, K_v_ 1.2, and K_v_ 1.4 expressed in human embryonic kidney cells

**DOI:** 10.3109/21556660.2013.791623

**Published:** 2013-04-02

**Authors:** Anthony Caggiano, Andrew Blight, Tom J. Parry

**Affiliations:** Acorda Therapeutics, Inc., Ardsley, NYUSA

**Keywords:** 4-Aminopyridine, Dalfampridine, Metabolites, Potassium channels

## Abstract

**Background:**

Dalfampridine (4-aminopyridine; 4-AP) is a potassium channel blocker that has been available in the United States as a treatment to improve walking in patients with multiple sclerosis. 4-AP is well-characterized *in vitro* with regard to inhibition of neuronal potassium channels, but the potential contribution of its metabolites to clinical activity has not been determined. This study evaluated the concentration–response of 4-AP and its two primary metabolites, 3-hydroxy-4-aminopyridine and 3-hydroxy-4-aminopyridine sulfate, for inhibition of the potassium channels K_v_ 1.1, K_v_ 1.2, and K_v_ 1.4, which are considered candidates for mediating effects of 4-AP on action potential conduction because of their presence in axonal membranes.

**Methods:**

Stable transfection of cDNA for K_v_ 1.1, K_v_ 1.2, and K_v_ 1.4 was performed into HEK293 cells, and colonies of cells containing each channel were selected and maintained under appropriate cell culture conditions. Electrophysiological measurements were performed using a patch-clamp technique in at least three cells for each concentration (50, 500, 5000, and 50,000 μM) of 4-AP and the two metabolites, with each cell acting as its own control. Concentration–response curves were constructed for 4-AP and each metabolite. Data were analyzed using nonlinear least-squares fit, and concentrations inhibiting the channels by 50% (IC_50_) were estimated.

**Results:**

4-AP induced similar concentration-dependent inhibition profiles of all three potassium channels, resulting in a narrow range of IC_50_ values across channels (242 µM to 399 µM). Across the three channels, the IC_50_ values of 3-hydroxy-4-aminopyridine and 3-hydroxy-4-aminopyridine sulfate were 1–2 orders of magnitude higher (less potent) than those of 4-AP.

**Conclusions:**

3-Hydroxy-4-aminopyridine and 3-hydroxy-4-aminopyridine sulfate demonstrated low *in vitro* potency for K_v_ 1.1, K_v_ 1.2, and K_v_ 1.4 inhibition, suggesting that these metabolites are unlikely to contribute to the positive pharmacodynamic effects of 4-AP. A limitation of this study is that while the metabolites were substantially less active at these representative potassium channels *in vitro*, the untested possibility exists that they may be active at one or more of the many other channel types that occur *in vivo*.

## Introduction

Dalfampridine (4-aminopyridine; 4-AP) is a potassium channel blocker that has been studied extensively in the laboratory and in the clinic. An extended-release formulation of the drug, dalfampridine extended release, is available in the United States for the treatment of walking impairment in patients with multiple sclerosis (MS)^1^. This formulation is also approved for this use in several other countries where it is known as prolonged-, sustained-, or modified release fampridine.

4-AP has been well-characterized *in vitro* with regard to inhibition of a wide range of neuronal potassium channels, although the mechanism of action of 4-AP in MS has not been clearly established. While concentrations that result in 50% inhibition (IC_50_) of these channels have been determined to be mostly in the millimolar range^2–4^, the average plasma concentration obtained with therapeutic dosing in clinical trials of the approved formulation ranged from 0.29 to 0.32 µM (27.6–30.2 *ng*/mL)^5,6^, which is 3–4 orders of magnitude lower than the typical concentrations used in *in vitro* laboratory studies to block potassium currents. However, the putative mechanism of action for its clinical effects is the relief of conduction block in demyelinated axons^7^, although it may also act at presynaptic sites, potentially enhancing neurotransmission through delay of repolarization and increased influx of calcium^8,9^.

Several studies have described the pharmacokinetic characteristics of 4-AP in healthy volunteers and in the target population of people with MS^10–14^. In an excretion balance study in healthy volunteers using ^14^C-radiolabeled 4-AP, elimination was almost exclusively (96%) by the renal route^10^. Although recovery in urine was mainly as unchanged parent compound, two primary metabolites were initially identified as 2-hydroxy-4-aminopyridine and 3-hydroxy-4-aminopyridine based on approximate retention times using high performance liquid chromatography. Further characterization using established reference standards showed that the human metabolites of 4-AP were 3-hydroxy-4-aminopyridine and 3-hydroxy-4-aminopyridine sulfate, the latter as a result of sulfate conjugation, and that they accounted for <10% of urinary excretion^15^. However, the potential contribution of these metabolites to the clinical activity of dalfampridine has not been determined.

Whereas K_v_ 1.1 and K_v_ 1.2 are voltage-gated potassium channels of the delayed rectifier type, K_v_ 1.4 is a fast inactivating channel of the A-type (Table 1)^16^. These channels were considered suitable for evaluating the relative potency of the parent drug and its metabolites, and they are considered candidates for mediating effects of 4-AP on action potential conduction because of their presence in axonal membranes; K_v_ 1.1, K_v_ 1.2 and K_v_ 1.4 are relevant components of axonal membrane heterotetrameric channels, and K_v_ 1.4 is also a homotetrameric synaptic membrane channel. However, the IC_50_ for these channels *in vitro* is much higher than the effective plasma concentration achieved with dalfampridine treatment. Therefore, the purpose of this study was to evaluate the concentration–response of 4-AP and its two primary metabolites for inhibition of three of the most common K_v_ channels, K_v_ 1.1, K_v_ 1.2, and K_v_ 1.4.

**Table 1.** Characteristics of K_v_ 1.1, 1.2, and 1.4 potassium channels (adapted with permission from Gutman *et al*.^16^).

Channel	IUPHAR IC_50_ for 4-AP, µM	Current type	Physiological function	Distribution
K_v_ 1.1	290	Voltage-gated, delayed rectifier channel in neurons and skeletal muscle	Maintaining membrane potential and modulating electrical excitability in neurons and muscle	Brain, heart, retina, skeletal muscle, islets
K_v_ 1.2	590	Voltage-gated, delayed rectifier channel	Maintaining membrane potential and modulating electrical excitability in neurons and muscle	Brain (pons, medulla, cerebellum, inferior colliculus > hippocampus, thalamus, cerebral cortex, superior colliculus > midbrain, corpus striatum, olfactory bulb; neurons associated with mechanoreception and proprioception), spinal cord, Schwann cells, atrium, ventricle, islet, retina, smooth muscle
K_v_ 1.4	13,000	Voltage-gated, A-type, fast-inactivating	Neuronal afterhypolarization	Brain (olfactory bulb, corpus striatum > hippocampus, superior and inferior colliculus > cerebral cortex, midbrain basal ganglia > pons/medulla), lung-carcinoid, skeletal muscle, heart, pancreatic islet

IUPHAR, International Union of Pharmacology; IC_50_, concentration resulting in 50% inhibition; 4-AP, 4-aminopyridine.

## Methods

This study was conducted in accordance with good laboratory practice standards. All chemicals were obtained from Sigma-Aldrich (St. Louis, MI, USA) unless noted otherwise.

### Cell cultures

Stable transfection of cDNA for each of the potassium channels was performed into HEK293 cells (American Type Culture Collection, Manassas, VA, USA), a human embryonic kidney cell line, as previously described^17^. After colony selection, the separate colonies of transfected HEK293 cells were cultured at 37°C in Dulbecco’s Modified Eagle Medium/Nutrient Mixture F-12 (D-MEM/F-12) supplemented with 10% fetal bovine serum, 100 U/mL penicillin G sodium, 100 µg/mL streptomycin sulfate and 500 µg/mL G418.

Cells used for electrophysiology were plated in plastic culture dishes. Before testing, the cells were washed twice with Hank’s balanced salt solution, treated with trypsin and re-suspended in culture medium at ∼1–1.5 × 10^6^ cells in 20 mL. Cells in suspension were allowed to recover for 1–3 hours with incubation at 37°C in a humidified 95% air/5% CO_2_ atmosphere. Immediately before electrophysiological measurement, the cells were washed in 4-[2-hydroxyethyl]-1-piperazineethanesulfonic acid (HEPES)-buffered physiological saline (HB-PS).

### Chemicals and reagents

The 4-AP was produced by Regis Technologies (Morton Grove, IL, USA), and the two metabolites, 3-hydroxy-4-aminopyridine and 3-hydroxy-4-aminopyridine sulfate, were synthesized by Dalton Pharma Services (Toronto, ON, Canada). Stock concentrations (50 mM) of 4-AP, 3-hydroxy-4-aminopyridine, and 3-hydroxy-4-aminopyridine sulfate were prepared in HB-PS physiological saline (87 mM NaCl, 4.0 mM KCl, 1.8 mM CaCl_2_, 1.0 mM MgCl_2_, 10 mM HEPES, 10 mM glucose; pH adjusted to 7.4 with HCl) and sonicated (Microson Ultrasonic Cell Disruptor, Misonix Inc., Farmingdale, NY, USA) at ambient room temperature for 5–10 minutes to facilitate dissolution. Test solutions were prepared fresh daily by diluting stock into HB-PS solution 137 mM NaCl, 4.0 mM KCl, 1.8 mM CaCl_2_, 1.0 mM MgCl_2_, 10 mM HEPES, 10 mM glucose (pH adjusted to 7.4 with NaOH). Four concentrations of each compound were evaluated: 50, 500, 5000, and 50,000 μM.

Test samples were applied at 5-minute intervals via disposable polyethylene micropipette tips to naïve cells. Each solution exchange was performed in quadruplicate, and consisted of aspiration and replacement of 45 µL of the total 50-µL volume. The duration of exposure to each sample concentration was 5 minutes.

### Electrophysiological measurements

Electrophysiological measurements were performed in at least three cells for each concentration using the PatchXpress system (Model 7000A, Molecular Devices LLC, Sunnyvale, CA, USA), at ambient temperature, with each cell acting as its own control. The methods for applying this commercially available patch-clamp system in the assessment of ion channels have been previously described^18^. In preparation for the recording, an intracellular solution consisting of 130 mM K-Asp, 5 mM MgCl_2_, 5 mM EGTA (ethylene glycol tetraacetic acid), 4 mM ATP (adenosine-5′-triphosphate), and 10 mM HEPES (pH to 7.2 with KOH) was loaded into the intracellular compartments of the Sealchip_16_ planar electrode (Aviva Biosciences, San Diego, CA, USA). Membrane currents were recorded using dual-channel patch clamp amplifiers, and before digitization, signals were low-pass filtered at one-fifth of the sampling frequency.

### Data analysis

Data acquisition and analysis were performed using pCLAMP software (Axon Instruments, Union City, CA, USA). Steady state was defined by the limiting constant rate of change with time (linear time dependence). The steady state before and after each application was used to calculate the percentage of current inhibited at each concentration. Results obtained from different cells were averaged and plotted as the mean ± standard deviation.

Concentration–response curves were constructed for each test compound, and data were fitted to the following equation:

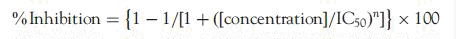

where % Inhibition represents the proportion of ion channel current inhibited at each concentration, IC_50_ is the concentration resulting in 50% inhibition, and *n* is the Hill coefficient. Nonlinear least-squares fits were performed with the Solver add-in for Excel 2000 (Microsoft Corporation, Redmond, WA, USA), and the IC_50_ was calculated. The kinetics of K_v_ 1.4 channel inactivation were analyzed with the Clampfit 9.2 program (Molecular Devices LLC).

## Results

**Figure 1.**
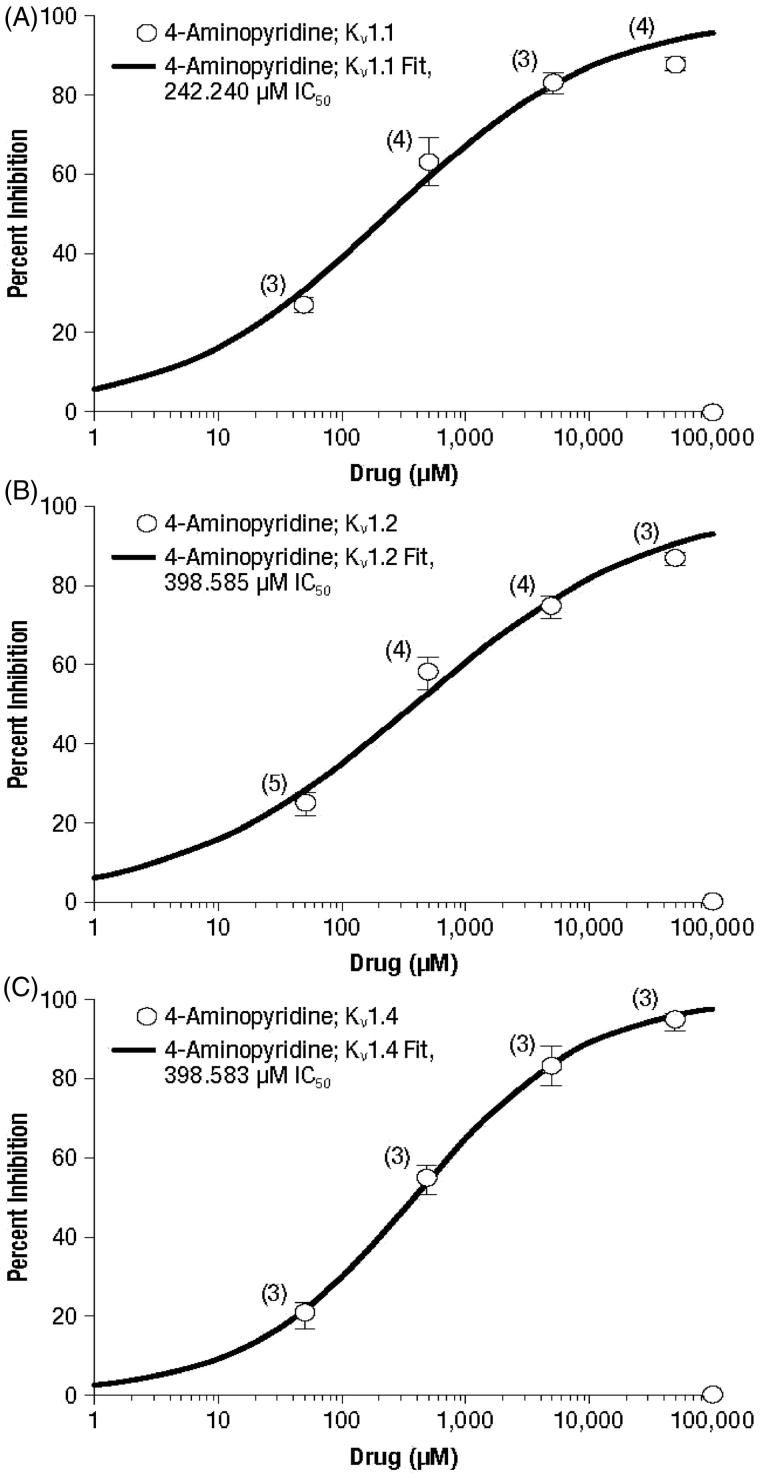
Concentration–response curves of 4-aminopyridine for the potassium channels K_v_ 1.1 (A), K_v_ 1.2 (B), and K_v_ 1.4 (C). Values represent mean percent inhibition ± standard deviation; numbers in parentheses represent the number of replicates.

**Figure 2.**
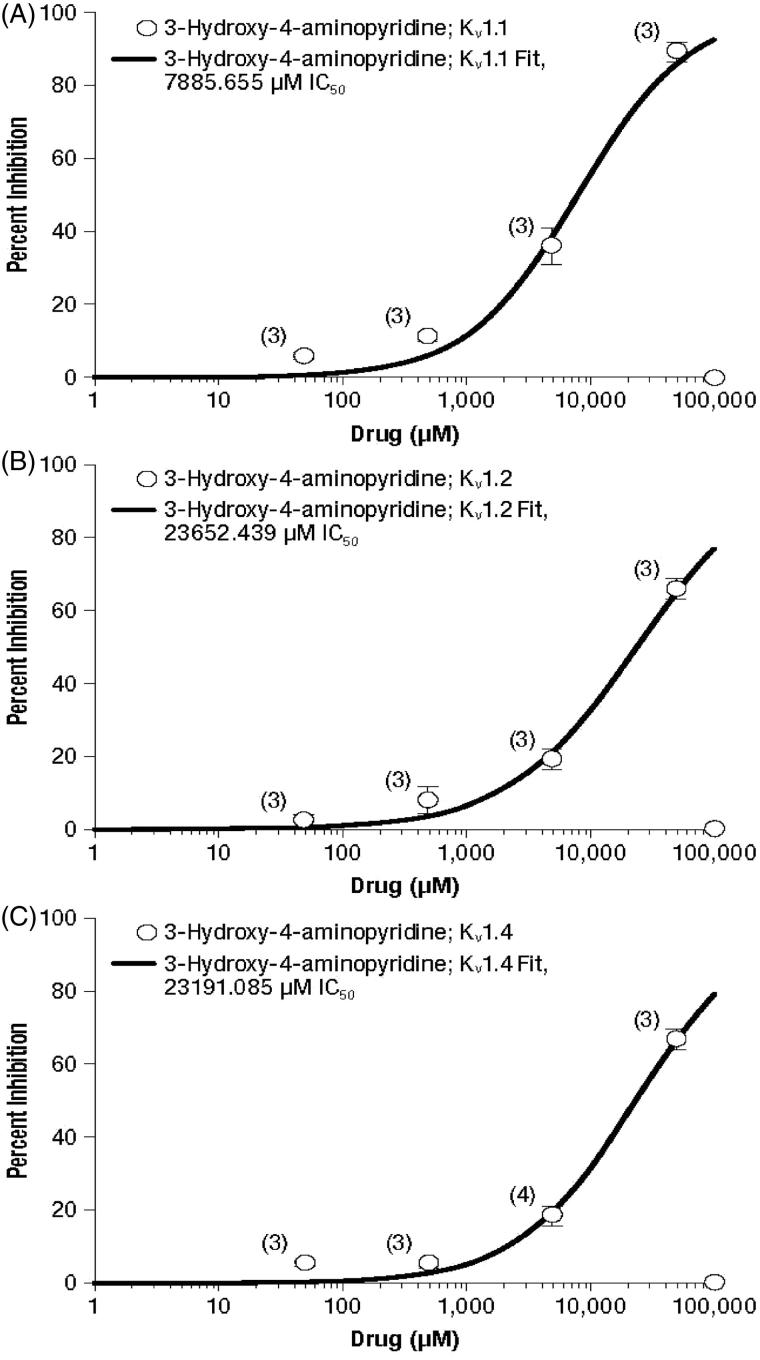
Concentration–response curves of 3-hydroxy-4-aminopyridine for the potassium channels K_v_ 1.1 (A), K_v_ 1.2 (B), and K_v_ 1.4 (C). Values represent mean percent inhibition ± standard deviation; numbers in parentheses represent the number of replicates.

**Figure 3.**
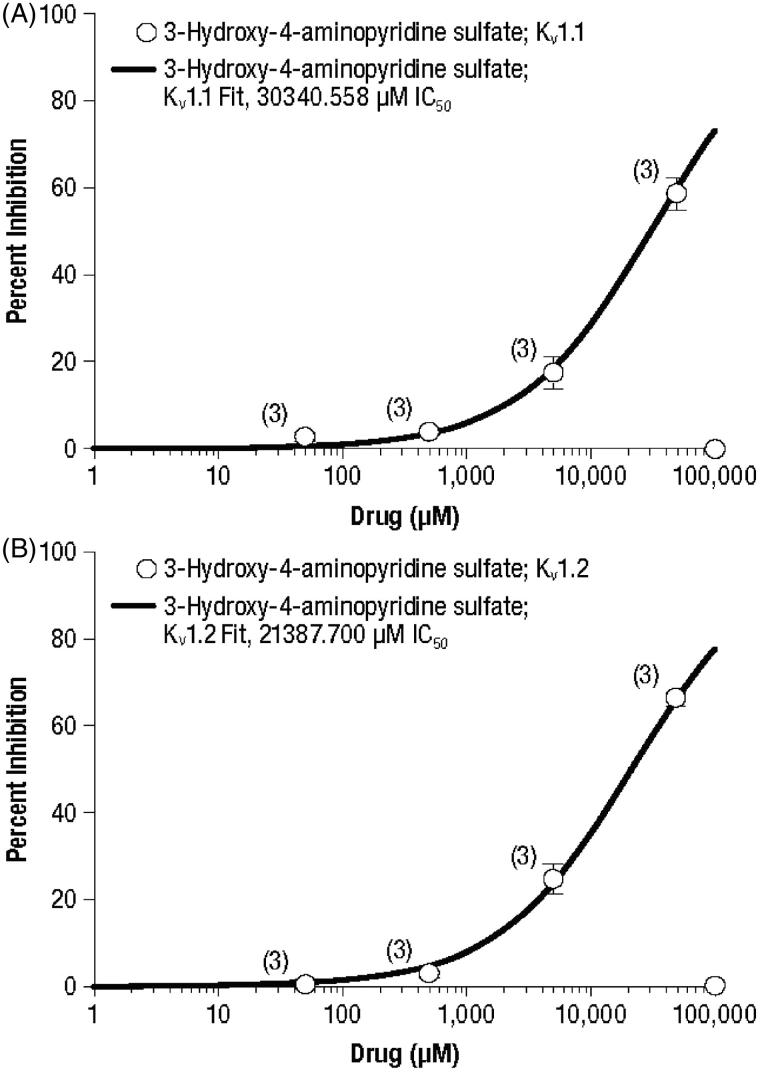
Concentration–response curves of 3-hydroxy-4-aminopyridine sulfate for the potassium channels K_v_ 1.1 (A) and K_v_ 1.2 (B). Values represent mean percent inhibition ± standard deviation; numbers in parentheses represent the number of replicates.

Concentration–response curves for inhibition of the three evaluated potassium channels are presented in Figures 1–3 for 4-AP and its metabolites 3-hydroxy-4-aminopyridine and 3-hydroxy-4-aminopyridine sulfate, respectively, and the calculated IC_50_ concentrations are summarized in Table 2.

**Table 2.** Concentrations of 4-aminopyridine and its two major metabolites that result in 50% inhibition of peak current of the recombinant potassium channels K_v_ 1.1, K_v_ 1.2, and K_v_ 1.4.

Test compound	IC_50_, µM		
	K_v_ 1.1	K_v_ 1.2	K_v_ 1.4
4-Aminopyridine	242	399	399
3-Hydroxy-4-aminopyridine	7,886	23,652	23,191
3-Hydroxy-4-aminopyridine sulfate	30,341	21,388	>50,000

IC_50_, concentration resulting in 50% inhibition.

As shown in Figure 1, 4-AP induced similar concentration-dependent inhibition profiles of all three potassium channels. The percent inhibition at each concentration was generally comparable among the channels, and ranged from 20.2% to 26.9% at 50 μM, 54.5–63.0% at 500 μM, 74.4–83.1% at 5000 μM, and 86.5–94.4% at 50,000 μM. This similarity resulted in a narrow range of estimated IC_50_ values across channels: 242 µM for K_v_ 1.1 and 399 µM for both K_v_ 1.2 and K_v_ 1.4 (Table 2).

In contrast, 3-hydroxy-4-aminopyridine demonstrated a greater potency for inhibition of K_v_ 1.1 than the other two channels (Figure 2). However, the IC_50_ values of 3-hydroxy-4-aminopyridine for K_v_ 1.1 (7886 µM), K_v_ 1.2 (23,652 µM) and K_v_ 1.4 (23,191 µM) were greater than one order of magnitude higher than those of 4-AP (Table 2), indicating less potency. Similarly, the IC_50_ values of 3-hydroxy-4-aminopyridine sulfate were approximately two orders of magnitude greater than those of 4-AP for K_v_ 1.1 and K_v_ 1.4 (Table 2). While the highest concentration of 3-hydroxy-4-aminopyridine sulfate (50,000 µM) resulted in 58.5% and 66.0% inhibition of K_v_ 1.1 and K_v_ 1.2, respectively (Figure 3), K_v_ 1.4 was only inhibited by 27%. Overall inhibition of K_v_ 1.4 by this metabolite was sufficiently low that a concentration–response curve was not derived.

Neither of the 4-AP metabolites demonstrated substantial effects on the kinetics of K_v_ 1.4 inactivation (Table 3). In contrast, inactivation was accelerated with 4-AP in a concentration-dependent manner, with enhancement of inactivation by 62.1% at 5000 μM; the percent change in inactivation was not determined at the highest 4-AP concentration of 50,000 μM.

**Table 3.** Effect of 4-aminopyridine and its two major metabolites on K_v_ 1.4 kinetics of inactivation. Positive values indicate acceleration of inactivation.

Test compound	Percent change in K_v_ 1.4 inactivation, mean ± standard deviation (n)			
	50 μM	500 μM	5000 μM	50,000 μM
4-Aminopyridine	6.8 ± 5.8 (3)	37.8 ± 2.8 (3)	62.1 ± 18.9 (3)	Not determined
3-Hydroxy-4-aminopyridine	−0.4 ± 7.4 (3)	1.7 ± 5.1 (3)	6.3 ± 10.0 (3)	20.3 ± 5.7 (3)
3-Hydroxy-4-aminopyridine sulfate	3.2 ± 19.7 (3)	−3.7 ± 7.6 (4)	1.9 ± 14.6 (3)	9.5 ± 15.0 (4)

## Discussion

This is the first study to evaluate the pharmacodynamic properties of the two major 4-AP metabolites against these potassium channels. Although the inhibitory potential of 4-AP for various potassium channels has previously been assessed, this study re-evaluated 4-AP for specific activity using stably transfected K_v_ 1.1, K_v_ 1.2, and K_v_ 1.4 channels.

The relevancy of the channels tested in this study was based on their presence in axonal membranes, with all three channels considered potential candidates for mediating the effects of 4-AP on action potential conduction since they are components of the juxtaparanodal heterotetrameric K_v_ channels. In particular, K_v_ 1.1 and K_v_ 1.2 have a distribution in the internodal membrane that is consistent with changes in 4-AP sensitivity following demyelination^3,19^. However, in this as well as in previous studies, the sensitivity of these channels to 4-AP appears to be too low to correspond to the clinically relevant, submicromolar concentrations achieved with therapeutic dosing in patients with MS^3^, and the maximization of clinical benefit at plasma concentrations less than 0.5 µM^20^, suggesting the participation of other potassium channels in these effects.

The IC_50_ values of 4-AP reported here for K_v_ 1.1 (242 μM) and K_v_ 1.2 (399 μM) were consistent with other studies reviewed by Judge *et al*.^3^ Therefore, given the higher concentrations needed to block these channels *in vitro* compared with the upper range of plasma concentrations of approximately 0.92 μM (87.3 ng/mL) reported with dalfampridine at the recommended daily dose of 10 mg twice daily in the clinical trials^5,6^, these channels do not appear to be likely candidates for the observed clinical effects.

The IC_50_ of 4-AP for K_v_ 1.4, 399 μM (37,506 ng/mL), was similar to that for K_v_ 1.1 and K_v_ 1.2. This is lower than has previously been reported (IC_50_ range of 647–13,000 μM)^3^. The reason for these differences is not clear but may be due to the source of the channels or the expression systems that were used in the previous studies. In particular, the study by Stühmer *et al*.^21^, which reported an IC_50_ of 13,000 μM, used cloned and sequenced cDNAs that were isolated from a rat cortex cDNA library, with channel function evaluated by expressing the cloned channels in *Xenopus laevis* oocytes. They then determined the concentration dependence of inhibition on outward current using whole cell recordings at 20 mV. In contrast, the study reported here relied on stable transfection of cDNA into HEK293 cells, and membrane currents were recorded using dual channel patch clamp amplifiers.

There are several potential reasons for the difference between therapeutic plasma concentrations and IC_50_ values for *in vitro* potassium-channel inhibition. The first is that it is possible that 4-AP is a potent inhibitor of one or more potassium channels that have not yet been identified. Given that 4-AP is clinically effective at systemic concentrations that are well below the IC_50_ values of the potassium channels tested so far^2^, it seems likely that this could be the case. Furthermore, how 4-AP concentrates in the microenvironment of a given K_v_ channel pore is not known, and thus plasma levels may not reflect what a transmembrane channel may experience. An alternative possibility is that *in vitro* assessment of inhibition of a cloned channel might not accurately represent what occurs in the native environment *in vivo*. In particular, given that potassium channel multimers are found *in vivo* that have not been tested using *in vitro* cloned channels^2^, this is also feasible.

It should also be considered that, given the ubiquity of potassium channels throughout the tissues of the body, and particularly the nervous system, if 4-AP were not highly selective for the particular channels in the target tissue (presumably the demyelinated axonal membrane), it would be unlikely to have any clinical utility, given all the additional potential effects it might have on other parts of the neuromuscular system and cell types. There are data indicating that the sensitivity of potassium channels increases by at least 10-fold in chronically damaged central nerve fibers in the context of experimental spinal cord injury^22^, and it is possible that channel expression in demyelinated plaques is highly specific and uniquely sensitive to the low concentrations of 4-AP achieved with the therapeutic dose.

For the three representative potassium channels tested, the IC_50_ values of the two primary metabolites of 4-AP (3-hydroxy-4-aminopyridine and 3-hydroxy-4-aminopyridine sulfate) were in every case more than 30-fold higher (less potent) than the parent compound. Thus, these metabolites appear to be unlikely to contribute significantly to the primary pharmacodynamic effect of 4-AP, at least through the potassium channels evaluated in this study.

It is important to recognize an important limitation of this study that warrants conservative interpretation of the results. While the metabolites were substantially less active at these representative potassium channels *in vitro*, there remains the possibility that they may be active at one or more of the many other channel types that have been described to occur *in vivo*.

## Conclusion

Compared with the parent drug, the two primary metabolites of 4-AP demonstrate low potency for blockade of the representative potassium channels K_v_ 1.1, K_v_ 1.2, and K_v_ 1.4 *in vitro*. Thus, these metabolites are unlikely to contribute to the positive pharmacodynamic effects of 4-AP that may be observed during treatment in patients with MS.
